# Supplementary material for: Biomimetic cardiac tissue culture model (CTCM) to emulate cardiac physiology and pathophysiology ex vivo
Source: Commun Biol. 2022 Sep 9;5:934. doi: 10.1038/s42003-022-03919-3 (PMC9463130; doi:10.1038/s42003-022-03919-3)
Supplement: Supplementary file 2 — Supplementary Information [file 42003_2022_3919_MOESM2_ESM.pdf]

Supplementary Information

Supplementary Fig 1

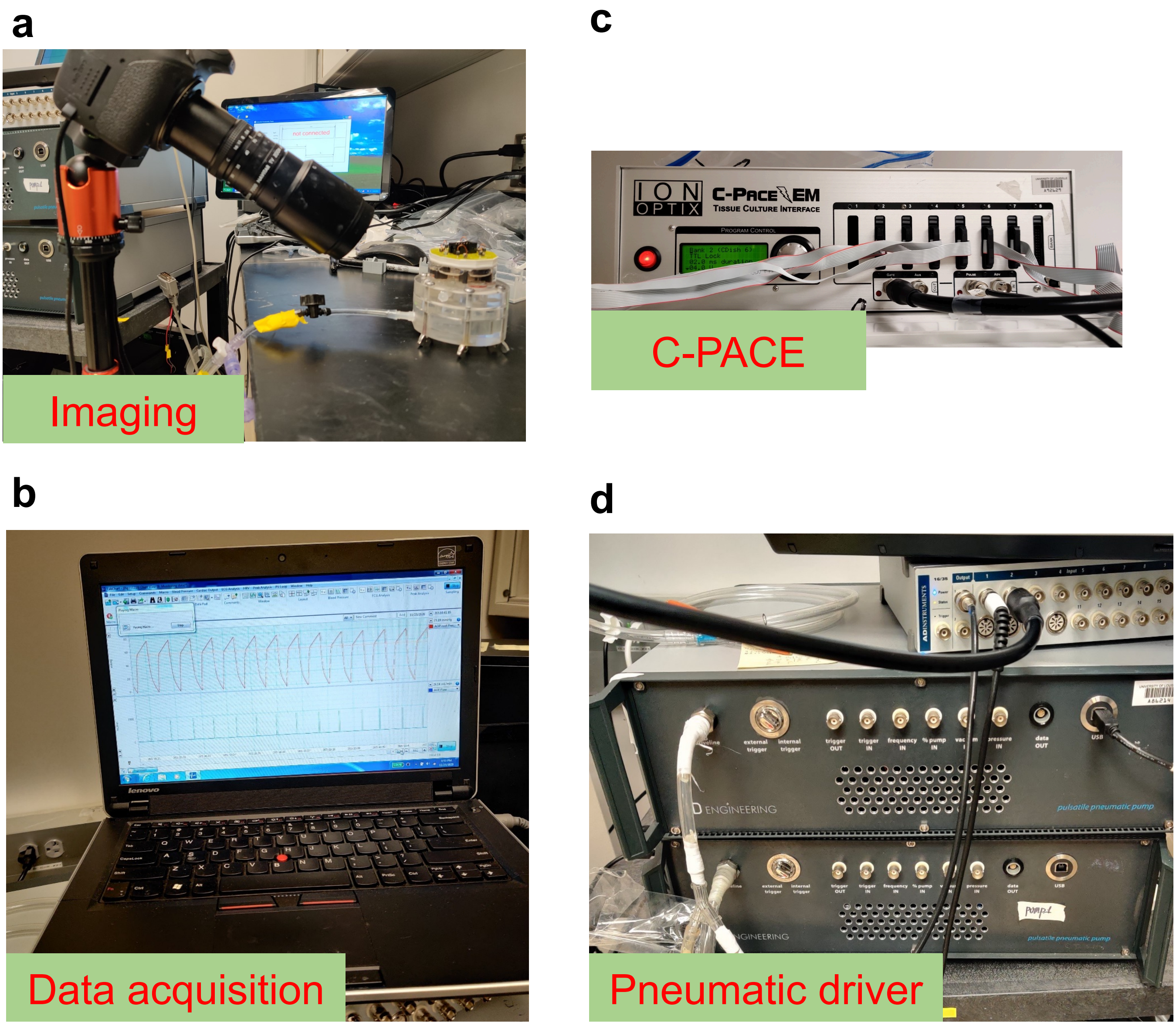

Supplementary Figure 1. CTCM device control systems and custom camera setup. (a) Custom camera setup to obtain high-resolution videos to characterize the tissue movements and stretches. **(b)** LabChart software showing data acquisition system for pressure probe sensor and electrical stimulation. **(c)** C-PACE device is used to induce electrical stimulation. **(d)** Pneumatic drivers are used to controlling air pressure within the air chamber.

Supplementary Fig 2

a

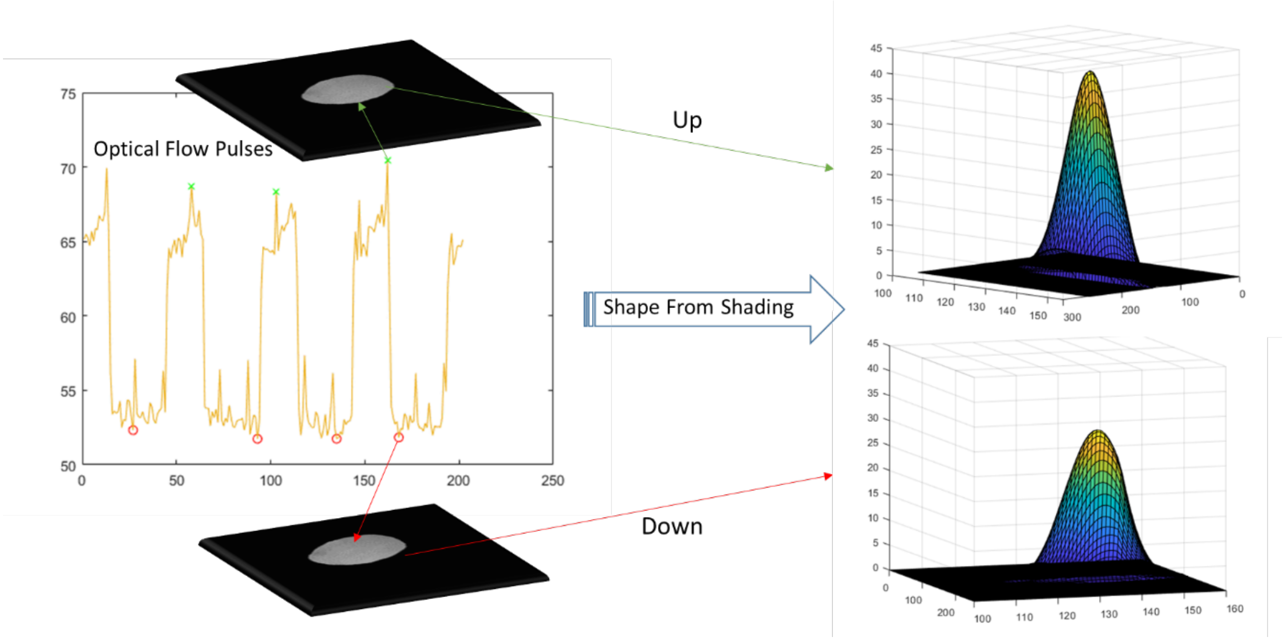

b

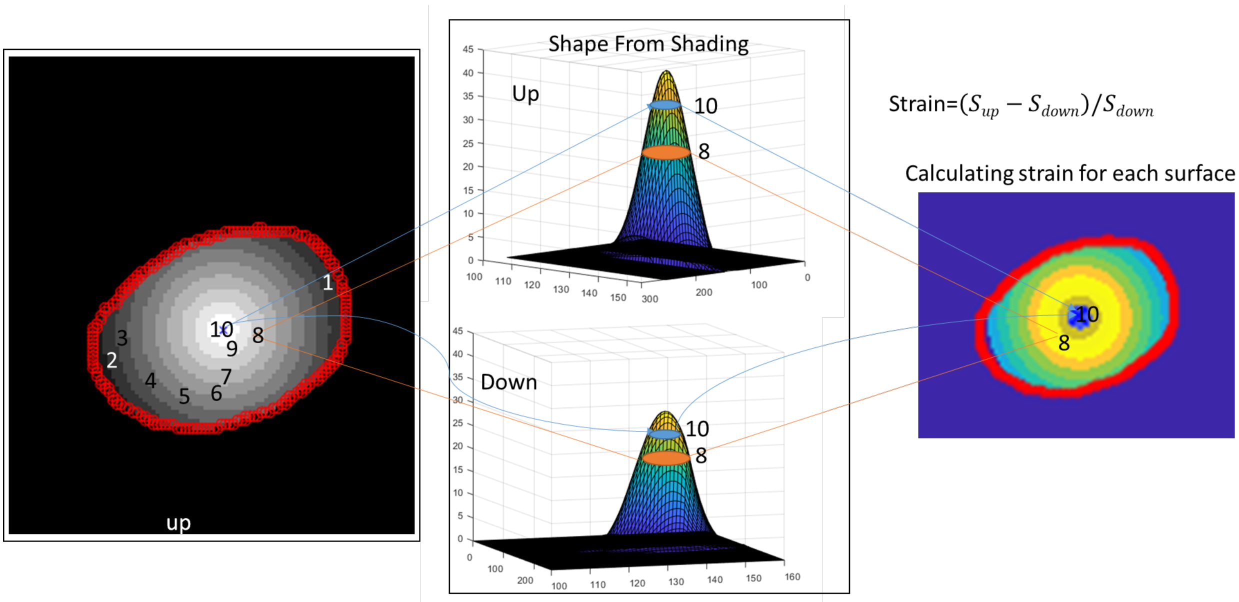

Supplementary Figure 2. Strain assessment in heart slices: (a) Application of Sahde from Shaping algorithm to build the 3D movement according to the peak movements. **(b)** segmentation of the 10 circular areas within the heart slice to determine the strain in each contour.

Supplementary Fig 3

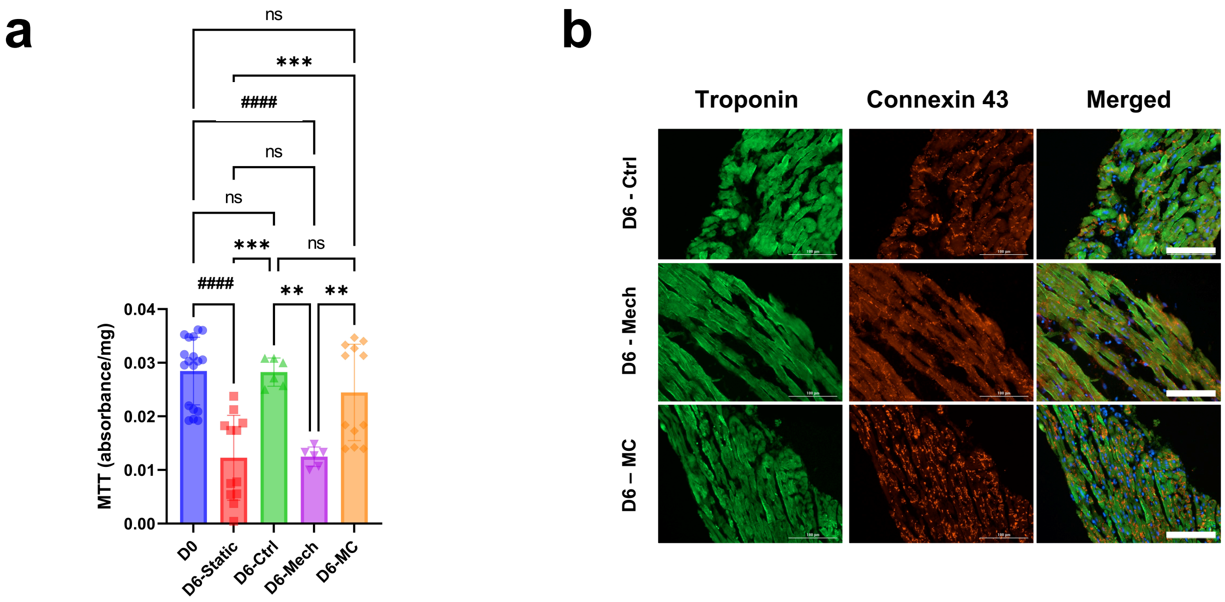

Supplementary Figure 3. Combination of electrical and mechanical stimulation resulted in the best tissue viability and structural integrity at 6 days. (a) Bar graph shows quantification of the MTT viability of fresh heart slices (D0) and heart slices cultured for 6 days in static biomimetic culture system with no electrical stimulation (D6 Static), static biomimetic culture system with electrical stimulation (D6 Ctrl), CTCM without electrical stimulation (D6 Mech), and CTCM with electrical stimulation (D6 MC) (n=18 (D0), 12 (D6-Static and D6-MC), 6 (D6-Ctrl, D6-Mech) from different pigs, one way ANOVA test is performed; ####p<0.0001 compared to D0, ***p<0.001 compared to D6 Static, and **p<0.01 compared to D6 Ctrl and D6 Mech). **(b)** Representative immunofluorescence images for troponin-T (green), connexin 43 (red), and DAPI (blue) of day 6 culture conditions (Scale bare=100μm). Error bars are representative of the Mean±SD.

Supplementary Figure 4

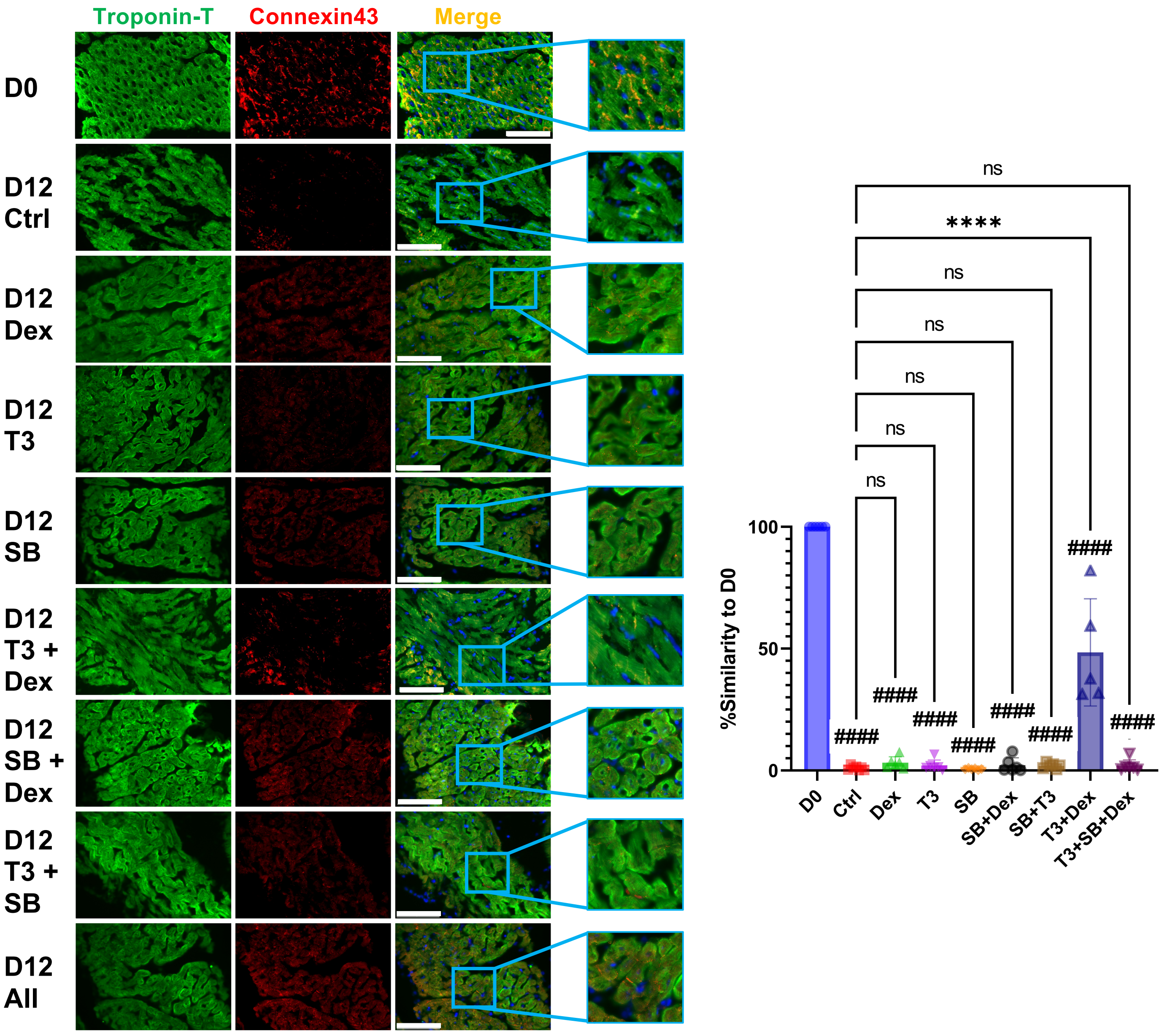

Supplementary Figure 4. Small molecule drug screening revealed that the combination of T3 and Dex improved heart slice structural integrity. Representative immunofluorescence images for troponin-T (green), connexin 43 (red), and DAPI (blue) for freshly isolated heart slices (D0) or heart slices cultured for 12 days under static conditions with standard media (Ctrl), 100nM tri-iodothyronine (T3), 1uM Dexamethasone (Dex) or 2.5uM SB431542 (SB) (Scale bare=100µm). Artificial intelligence quantification of the heart tissue structural integrity (n=7 (D0 and Ctrl), 5 (Dex, T3, SB, SB+Dex, SB+T3, T3+Dex, and T3+SB+Dex) slices each from different pig, one way ANOVA test is performed; ####p<0.0001 compared to D0 and ****p<0.0001 compared to D12 Ctrl). Error bars are representative of the Mean±SD.

Supplementary Figure 5

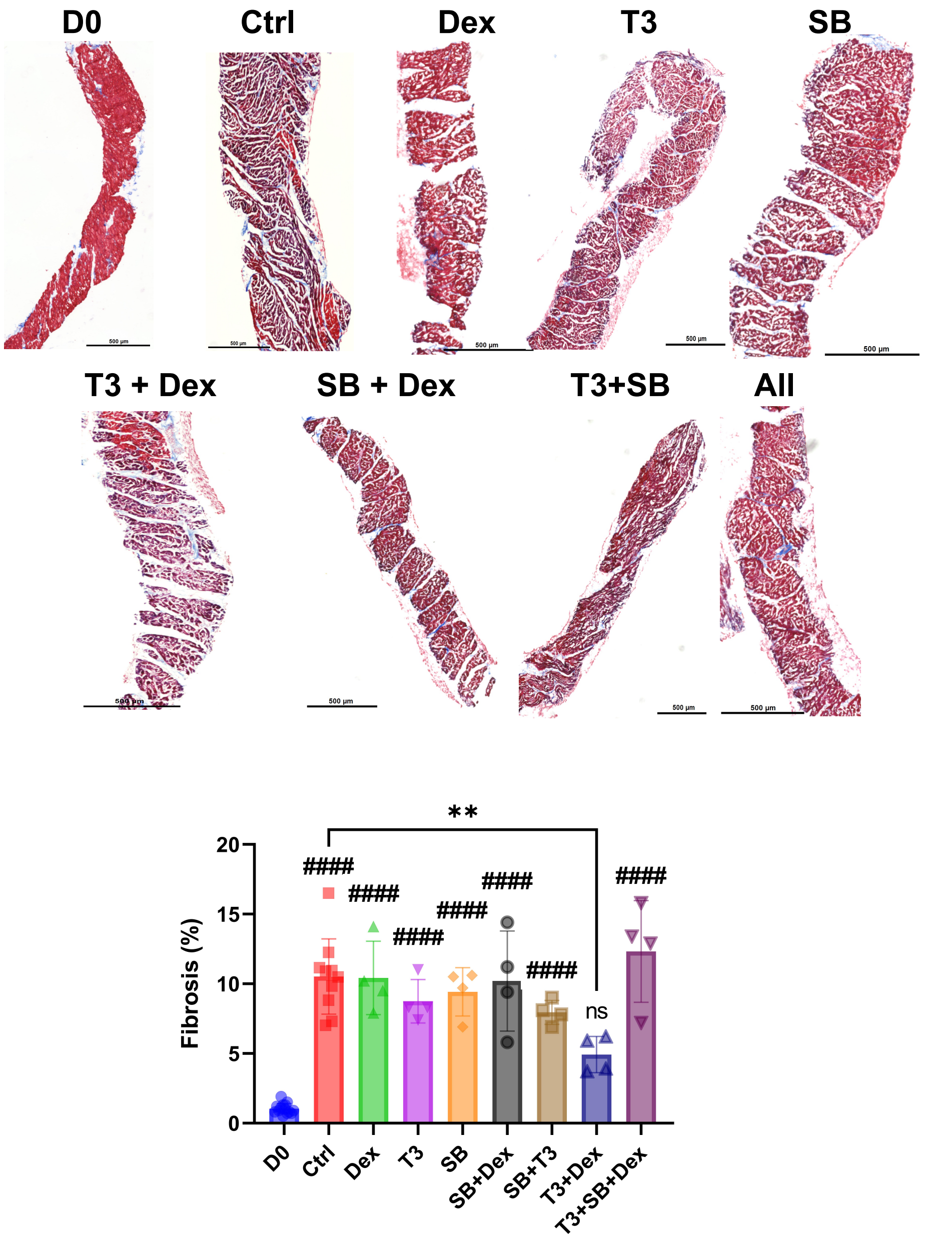

Supplementary Figure 5. Small molecule drug screening revealed that the combination of T3 and Dex showed reduced fibrosis formation. Representative Masson's trichrome images (Scale bare=500µm) for freshly isolated heart slices (D0) or heart slices cultured for 12 days under static conditions with standard media (Ctrl), 100nM tri-iodothyronine (T3), 1uM Dexamethasone (Dex) or 2.5uM SB431542 (SB). Bar graph shows the quantification of the fibrosis in heart slices (n=10 (D0 and Ctrl), 4 (Dex, T3, SB, SB+Dex, SB+T3, T3+Dex, and T3+SB+Dex) slices each from different pig, one way ANOVA test is performed; ####p<0.0001 compared to D0 and **p<0.01 compared to D12 Ctrl). Error bars are representative of the Mean±SD.

Supplementary Figure 6

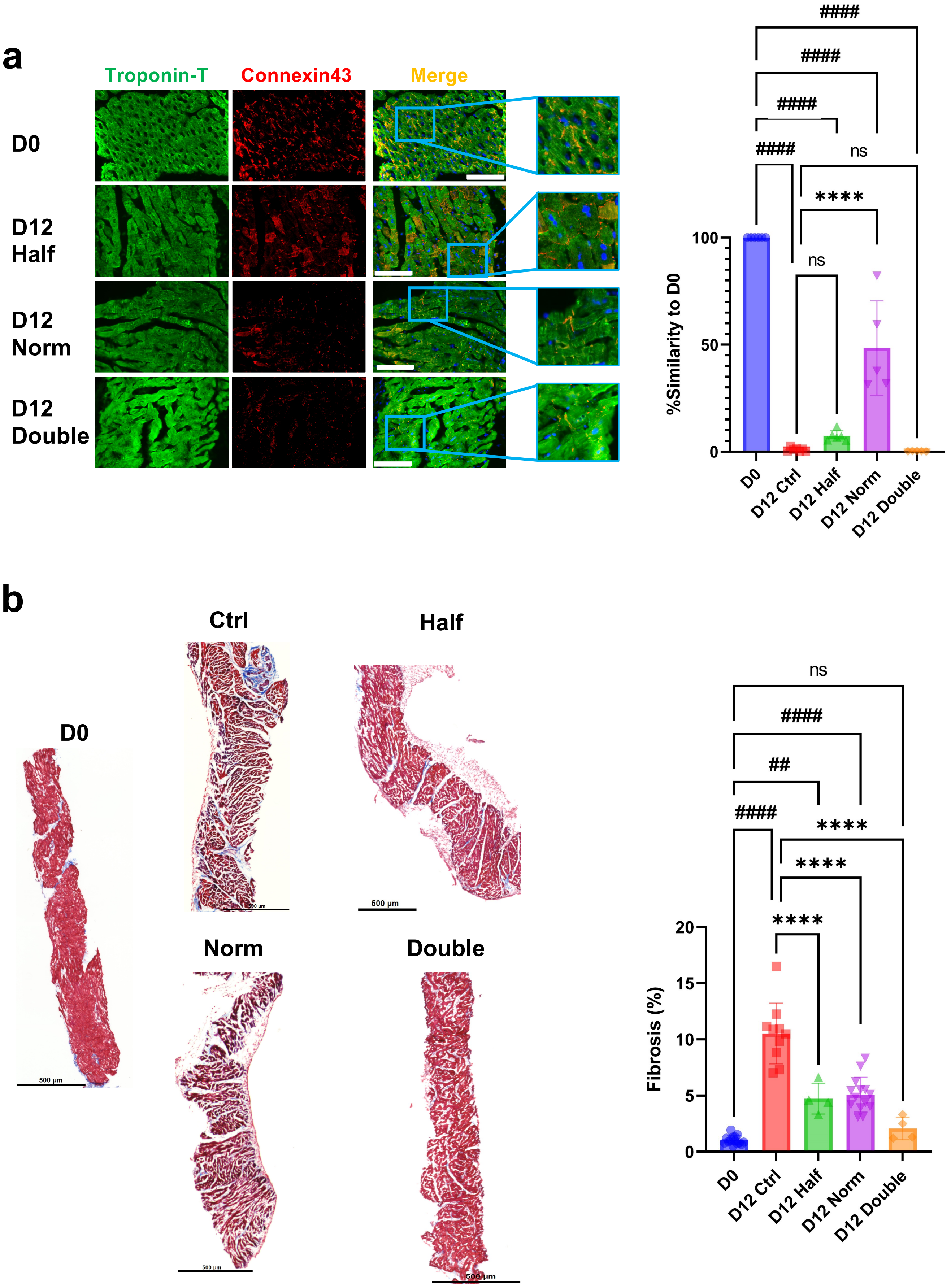

Supplementary Figure 6. 100 nM T3 and and 1μM Dex demonstrated the best improvement in tissue slice structural integrity. (a) Representative immunofluorescence images showing troponin-T (green) and connexin 43 (red) (Scale bare=100μm) demonstrating that the concentration of 100 nM T3 + 1uM Dex (Norm) resulted in a significantly better similarity to D0, compared to half dosages (Half) or double dosages (Double) of the T3 and Dex and the same day static culture control (Ctrl) (n=7 (D0 and D12 Ctrl), 5 (D12 Half, D12 Norm and D12 Double) slices/group from different pigs, one way ANOVA test is performed; ####p<0.0001 compared to D0 and ****p<0.0001 compared to D12 Ctrl) **(b)** Representative Masson's trichrome images (Scale bare=500μm) demonstrated a significant reduction of fibrosis formation with any dosage compared to same day control (n=14 (D0, D12 Ctrl, and D12 Norm), 4 (D12 Half, and D12 Double) slices/group from different pigs, one way ANOVA test is performed; ####p<0.0001 compared to D0 and ****p<0.0001 compared to D12 Ctrl). Error bars are representative of the Mean±SD.

Supplementary Figure 7

a **RNAseq differentially expressed genes between D0 vs D12 MT**

gene_name	gene_id	Fold D0 vs D12MT	log2 Fold D0 vs D12MT	P D0 vs D12MT	Rank	padj	padj (-log10)
LARS1	ENSSSCG00000014411	0.656222577	-0.607742865	0.000671558	28	0.049503437	1.305364646
H3-3A	ENSSSCG00000023971	0.693629708	-0.527762405	0.000676351	29	0.048137565	1.317515879
AIMP1	ENSSSCG00000024168	0.591906127	-0.756559704	0.000680511	30	0.046819162	1.32957636
ACP4	ENSSSCG00000024736	0.426109675	-1.230703285	0.000681436	31	0.045370473	1.343226695
RGL1	ENSSSCG00000015563	1.359906918	0.443507907	0.000681592	32	0.043962714	1.356915505
CYB5A	ENSSSCG00000004875	0.452448051	-1.144175941	0.000720379	33	0.045056457	1.346242965
PARP12	ENSSSCG00000016502	4.92866319	2.301196395	0.000737458	34	0.044768022	1.349032091
UQCRC2	ENSSSCG00000007845	0.727894632	-0.458198471	0.000767918	35	0.045285242	1.344043306
PKDCC	ENSSSCG00000008468	5.901218976	2.561012993	0.00078657	36	0.045096705	1.345855187
-	ENSSSCG00000037399	0.365075377	-1.453733728	0.00080162	37	0.044717396	1.34952349
ECHDC1	ENSSSCG00000004216	0.806135221	-0.310906238	0.000820961	38	0.044591163	1.350751201
LSM11	ENSSSCG00000038248	56.1341651	5.810807205	0.000853706	39	0.045180739	1.345046672
CYGB	ENSSSCG00000017181	2.128773815	1.09002267	0.000954347	40	0.049244292	1.307644101
PDIA3	ENSSSCG00000004700	1.079368959	0.110188103	0.001572367	65	0.049928711	1.301649643
TBC1D9	ENSSSCG00000032741	1.663599966	0.734308561	0.001578087	66	0.049351087	1.306703281
NR4A3	ENSSSCG00000005385	0.286789911	-1.801933823	0.001606246	67	0.049481963	1.305553077

b

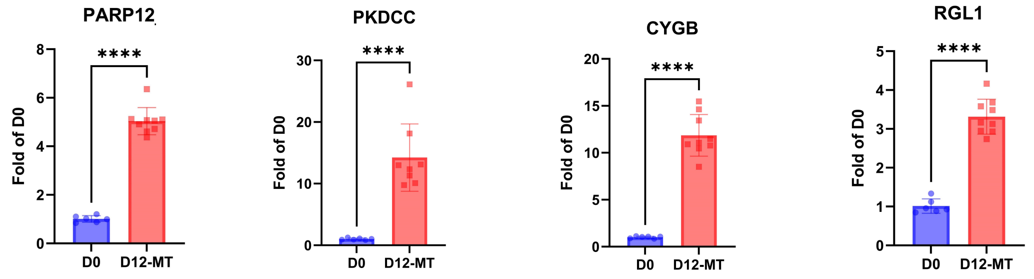

c

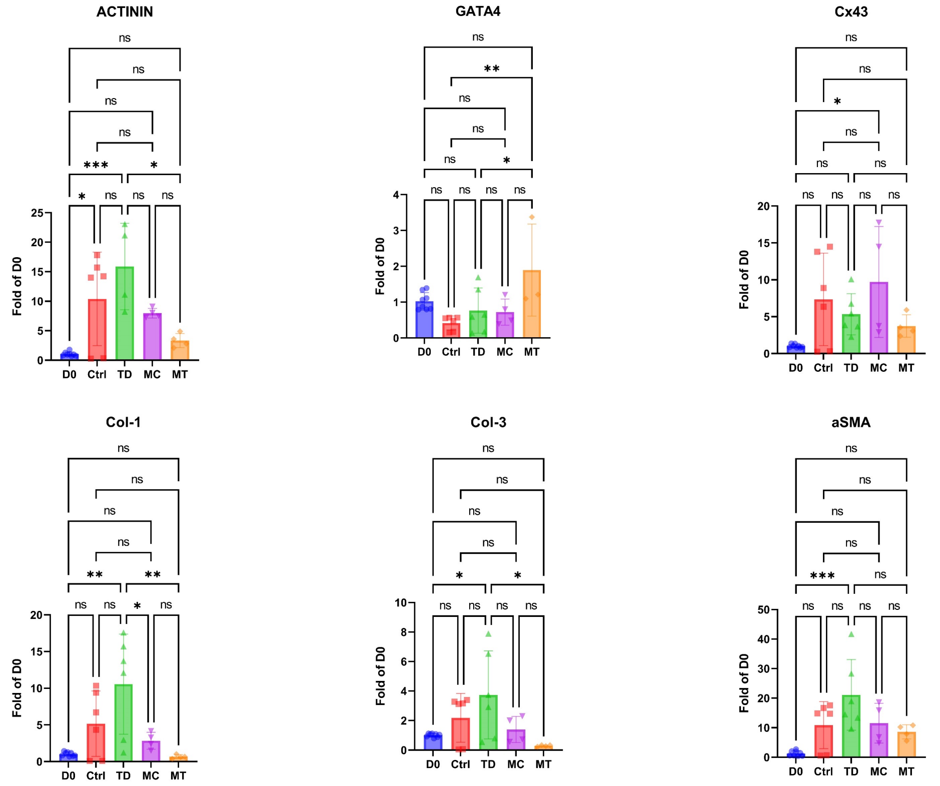

Supplementary Figure 7. Validation of RNAseq data using qRT-PCR. (a) Table listing the 16 differentially expressed genes between fresh tissue slices and day 12 MT from RNAseq. **(b)** bar graphs showing the RNA expression levels of the 4 selected genes from RNAseq of differentially expressed genes between day 0 and day 12 MT tissue using qRT-PCR. (n=6 (D0), 9 (D12MT), ****p<0.0001) **(c)** quantification of gene expression using qRT-PCR of major cardiac and fibroblast genes for fresh tissue (D0) and day 12 conditions. (n=8 (D0), 6 (Ctrl, and TD), 4 (MC and MT), ***p<0.001, **p<0.01, *p<0.05). Error bars are representative of the Mean±SD.

Supplementary Figure 8

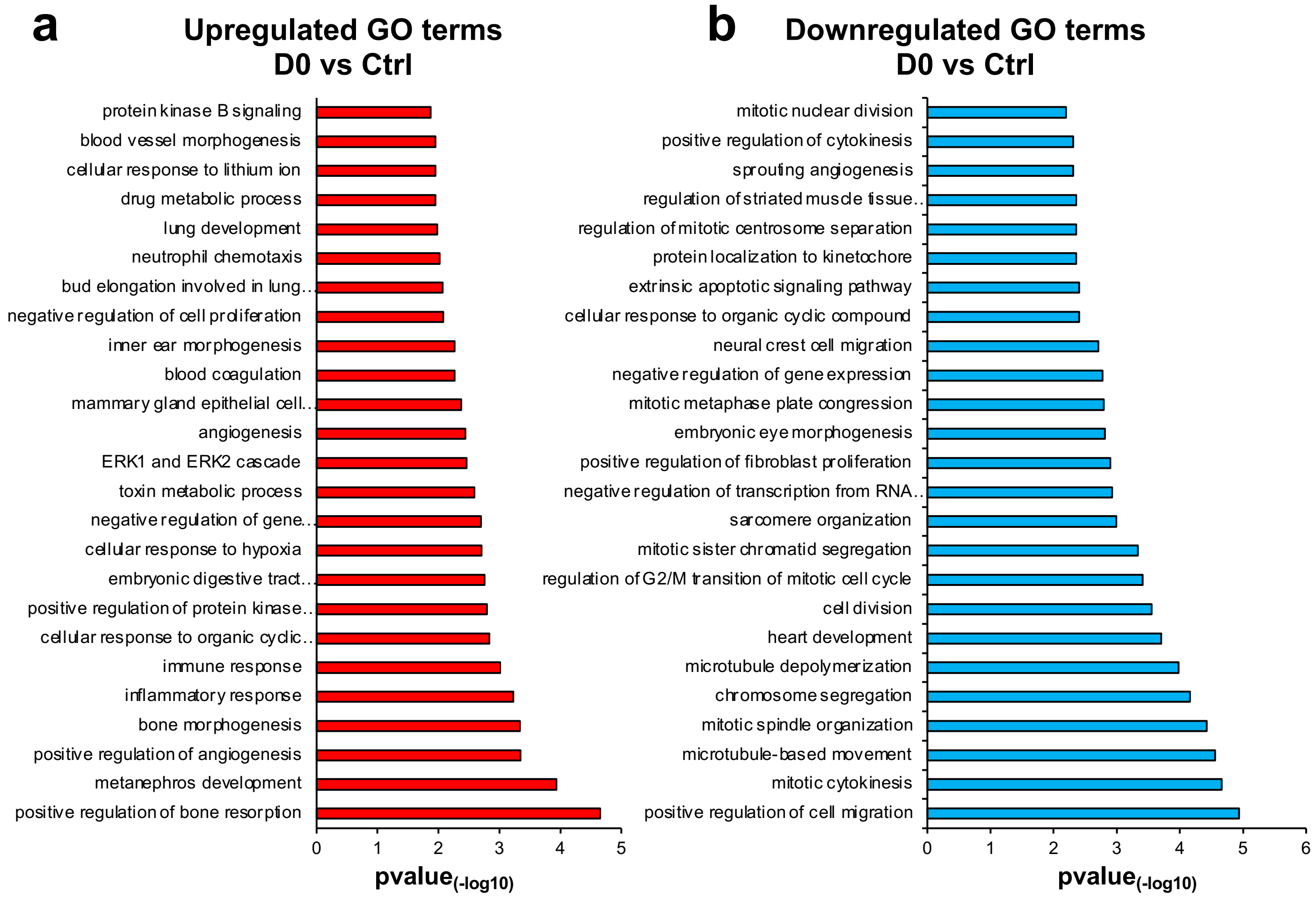

Supplementary Figure 8. Gene ontology (GO) for differentially gene expression between control (D12) and D0 shows upregulation of inflammatory gene and downregulation of cardiac genes . (a) Top upregulated GO terms showing a significant upregulation of inflammatory genes. **(b)** Top downregulated GO terms showing a significant downregulation of cardiac and cell cycle genes.

Supplementary Figure 9

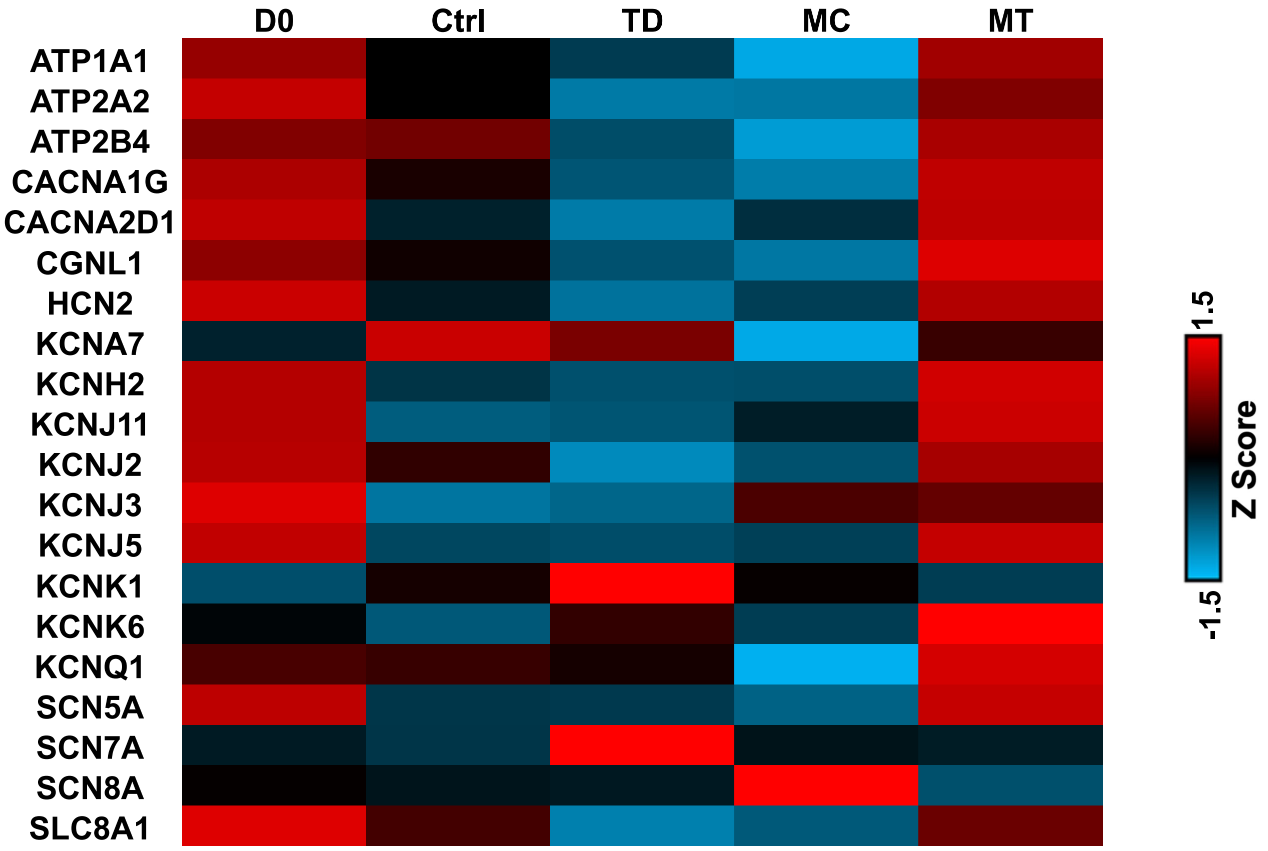

Supplementary Figure 9. Heatmap for the gene expression of the ion channel encoding genes
