# Supplementary material for: Biomimetic cardiac tissue culture model (CTCM) to emulate cardiac physiology and pathophysiology ex vivo
Source: Commun Biol. 2022 Sep 9;5:934. doi: 10.1038/s42003-022-03919-3 (PMC9463130; doi:10.1038/s42003-022-03919-3)
Supplement: Supplementary file 3 — Description of Additional Supplementary Data [file 42003_2022_3919_MOESM3_ESM.docx]

**Description of Additional Supplementary Files**

**File name:** Supplementary Data 1

**Description:** The source data behind the graphs in the paper and the RNAseq

**File name:** Supplementary software code

**Description:** The software codes used in the analysis

**File name:** Supplementary movie 1

**Description:** CTCM tissue movement during cardiac cycle
