# Supplementary material for: Biomimetic cardiac tissue culture model (CTCM) to emulate cardiac physiology and pathophysiology ex vivo
Source: Commun Biol. 2022 Sep 9;5:934. doi: 10.1038/s42003-022-03919-3 (PMC9463130; doi:10.1038/s42003-022-03919-3)
Supplement: Supplementary file 6 — Supplementary software code [file 42003_2022_3919_MOESM6_ESM.docx]

### Quantification of tissue stretch during the cycle

To calculate the peak movement of the heart slices, frame-by-frame analysis was performed manually using Image J, (NIH, Washington DC, USA). The center point of the tissue was tracked manually at each frame and a peak trace was calculated with the aid of a reference length (the ring diameter, 7mm) and the camera incline. Pixel length measurements were converted using the following equation

$$\begin{aligned} S=\frac{L_{m} \left[ pixels \right]}{L_{ref}\left[ pixels \right]\times\cos\left( \emptyset\right)}\times7 \left[ mm \right]\#\left( 1 \right) \end{aligned}$$

Where $S$ is the tissue peak movement in mm, $L_{m}$ is the measured peak movement in pixels, $L_{ref}$ is the ring diameter as in the frame in pixels, and $\emptyset$ is the camera inclination angle in degrees. Tissue movement was approximated a membrane movement that follows the equation [1]

$$\begin{aligned} y\left( r \right)=S \left( 1-\left( \frac{r}{R} \right)^{2} \right)^{2}\#\left( 2 \right) \end{aligned}$$

Where $y$is the upwards deflection of the tissue at distance $r$ from the center, $S$ is the tissue peak movement at any frame, $r$is the distance from the center, and $R$ is the tissue diameter.

Once the peak movement was determined at each frame, %area stretch was calculated using surface rotation of equation 2

$$\begin{aligned} \%stretch=({\pi R}^{2}-\int_{0}^{R} 2\pi r\sqrt{1+\left( \frac{dy}{dr} \right)^{2}}dr)/{\pi R}^{2}\times100 \#\left( 3 \right) \end{aligned}$$

These calculations were performed using custom software written in MATLAB.

**MATLAB code for Stretch Assessment**

%%%% Calculation of %Heart Slice %stretch by area%%%%%

%%%% Under sampling to 25 fps to allow for manual detection of a beat

%%%% dt = 0.04 s

%%%% ynorm is the center deflection calculated from normal condition

%%%% yOL is the center deflection calculated from overload condition

R=3.5; % heart slice radius

r = 0:0.001:R; %points along radius to calculate tissue deflection

for i=1:length(y0norm)

y0 = y0norm(i);

ynorm = y0.*(1-r.^2./(R^2)).^2; %tissue deflection in mm

yynorm = 2*y0.*(1-r.^2./(R^2)).*(-2*r/(R^2)); %dy/dr

ssnorm = 2*pi*r.*(1+yynorm.^2).^0.5; %term to be used to calculate integral for surface area of rotation

A_norm(i) = trapz(r,ssnorm);

end

for i=1:length(y0OL)

y0 = y0OL(i);

yOL= y0.*(1-r.^2./(R^2)).^2; %tissue deflection in mm

yyOL = 2*y0.*(1-r.^2./(R^2)).*(-2*r/(R^2)); %dy/dr

ssOL = 2*pi*r.*(1+yyOL.^2).^0.5; %term to be used to calculate integral for surface area of rotation

A_OL(i) = trapz(r,ssOL);

end

%stretch calculation

A0 = pi*R^2; %unstretched area

stretch_norm = 100 * (A_norm-A0)./A0; %for normal condition

stretch_OL = 100 * (A_OL-A0)./A0; %for overload condition

t = 0:0.04:length(y0norm)/25-0.04; %time base for plotting

plot(t,stretch_norm,t,stretch_OL) %plotting stretch

legend('Normal','Overload')

xlabel('Time (s)')

ylabel('%stretch')

**MATLAB Code for heart slice movement assessment**

**1 – Masking code**

% masking the stack of images 1 by 1

% choosing directory

% for j=0:12 % loop through days

close all

j=12;

n = int2str(j);

Day= ['D' n];

files = dir(fullfile('C:\Users '));

output = fullfile(' C:\Users ');

%open first image to add mask

img_to_mask = imread(files(3).name);

image_displayed = img_to_mask(:,:,(1:3));

imshow(image_displayed)

%create mask using the first image

roi = images.roi.AssistedFreehand;

draw(roi);

BW_mask_logic = createMask(roi); %Logic Mask

BW_mask = cast(BW_mask_logic, 'uint8'); %convert mask to uint8

% loop through the folder images and mask each

for i=1:length(files)-2

% if rem(i+2,2)~=0

image = imread(files(i+2).name);

image = image(:,:,(1:3));

Masked_image = BW_mask.*image;

baseFileName = sprintf('Image_%d.tif',i );

fullFileName = fullfile(output, baseFileName);

imwrite(Masked_image,fullFileName)

% end

end

% end

**2 – Data extraction**

clc

%10/21/21

%This code goes through a useful contraction signal and outputs:

%1)The mean contraction, 2)The peaks and valleys, 3)The cycle times

% for i=0:12

i=25;

start = 1; %start frame for analysis %input('What is the start index for peak and valley analysis?');

endtime = 353;%end frame for analysis %length(Contraction); %input('What is the end index for peak and valley analysis?');

n = int2str(i);

Day= ['D' n];

%creating a directory to access the contraction data

naming = ['C:\Users' Day '-Masked-Contr-Results'];

files = dir(fullfile(naming));

% close all

T= readtable('contraction30fps2');

time = (T.Var1)./1000;

Contraction = T.Var2;

% applying zero phase filter

d1 = designfilt('lowpassiir','FilterOrder',1, ...

'HalfPowerFrequency',0.4,'DesignMethod','butter');

cont = filtfilt(d1,Contraction); %filtered contraction

figure(1)

plot(cont)

grid on

% axis([7 inf 10 90])

xlabel('Frame')

ylabel('Contraction (a.u.)')

hold on

plot(Contraction)

hold off

der_cont = diff(cont);

% figure

% plot(der_cont)

%choosing section of interest

cont_ana = cont(start:endtime);

time_ana = time(start:endtime);

% Detrending the data to remove signal drift

[p,s,mu] = polyfit((1:numel(cont_ana))',cont_ana,20);

f_y = polyval(p,(1:numel(cont_ana))',[],mu);

cont_ana_trend=cont_ana;

% cont_ana = cont_ana-f_y;

figure(3)

plot(cont_ana)

hold on

plot(cont_ana)

plot(f_y)

hold off

%finding the peak

[peakValues, indexes_pea] = findpeaks(cont_ana,'MinPeakProminence',7);

figure(2)

plot(time_ana,cont_ana)

hold on

scatter(time_ana(indexes_pea),peakValues)

%refining the results using visual cues from derivative

cycle_time = diff(time_ana(indexes_pea));

% %finding valleys

invertedcont_ana = max(cont_ana) - cont_ana;

[~, indexes_val] = findpeaks(invertedcont_ana,'MinPeakProminence',7);

valleyValues = cont_ana(indexes_val);

scatter(time_ana(indexes_val),valleyValues)

%statistics

up = mean(peakValues);

std_up = std(peakValues);

down= mean(valleyValues);

std_down = std(valleyValues);

% yline(up)

% yline(down)

figurename = ['D' n '_contraction.png'];

saveas(gcf,figurename)

hold off

CONTRACTION=min(peakValues)-max(valleyValues);%up-down;

Regularity= [mean(cycle_time);std(cycle_time)];

% save(['Contraction ' Day],'CONTRACTION')

save(['Contraction' Day],'peakValues','valleyValues','CONTRACTION','Contraction','cycle_time','Regularity','cont_ana')

**3 – Data analysis**

close all

clear

i =0;

l = int2str(i);

load('FileName.mat')

figure(12)

% plot(cont_ana2)

hold on

plot(cont_ana)

d1 = designfilt('lowpassiir','FilterOrder',2, ...

'HalfPowerFrequency',0.4,'DesignMethod','butter');

%data to be extracted: 1-cycle time, 2-contraction time, 3-relaxation time,

%4 - contraction velocity

%%

fps = 30;

%%

%FIND DERIVATIVE TO CALCULATE THE CONTRACTION VELOCITY

t=0:1/fps:length(cont_ana)*1/fps-1/fps; % time

der = diff(cont_ana); %movement rate

speed = [der(1).*fps ; der.*fps]; %converting from frames to seconds. (a.u/sec)

%peaks for velocity

[peakValues2, indexes_pea2] = findpeaks(speed,'MinPeakProminence',100);

%valleys for velocity

inverted_speed = max(speed) - speed;

[~, indexes_val2] = findpeaks(inverted_speed,'MinPeakProminence',100);

valleyValues2 = speed(indexes_val2);

%getting peak contraction and relaxation speed

peak_relaxation_speed = mean(peakValues2);

peak_contraction_speed = mean(valleyValues2);

figure(11)

plot(t,speed)

hold on

scatter(t(indexes_pea2),peakValues2)

scatter(t(indexes_val2),valleyValues2)

%peaks for movement

[peakValues, indexes_pea] = findpeaks(cont_ana,'MinPeakProminence',7);

%valleys for movement

inverted_cont_ana = max(cont_ana) - cont_ana;

[~, indexes_val] = findpeaks(inverted_cont_ana,'MinPeakProminence',7);

valleyValues = cont_ana(indexes_val);

Amplitude = mean(peakValues)-mean(valleyValues);

%if you have more valleys than peaks

Amps_IND_amps = peakValues-valleyValues(1:length(peakValues));

%if you have more peaks than valleys

% Amps_IND_amps = peakValues(1:length(valleyValues))-valleyValues;

figure(1)

subplot(2,1,1)

plot(t,cont_ana,'LineWidth',1.5)

title('D6 - NORM Slices')

ylabel('Heart slice movement (a.u)','FontSize',12)

box off

hold on

scatter(t(indexes_pea),peakValues)

scatter(t(indexes_val),valleyValues)

axis([0 4.16667 -inf inf])

hold off

subplot(2,1,2)

plot(t,speed,'LineWidth',1.5)

ylabel('movement speed (a.u)/sec','FontSize',12)

xlabel('Time (s)','FontSize',12)

hold on

% scatter(t(indexes_val2),valleyValues2)

% scatter(t(indexes_pea2),peakValues2)

axis([0 4.1667 -inf inf])

hold off

box off

%cycle extraction from the speed

first_peak2 = indexes_pea2(2); %position of first peak for analysis

last_peak2 = indexes_pea2(end);%position of last peak for analysis

adjusted_peak2 = indexes_pea2(indexes_pea2>=first_peak2&indexes_pea2<=last_peak2);%signal for analysis starts with peak and ends with peak

%selecting valleys between peaks

adjusted_valley2 = indexes_val2(indexes_val2>first_peak2&indexes_val2<last_peak2);

B_cycle_time2 = diff(adjusted_peak2).*1/fps; %cycle times in seconds

B_relaxation_time2 =abs((adjusted_valley2-adjusted_peak2(2:end)).*1/fps);

B_contraction_time2 = abs((adjusted_peak2(1:end-1)-adjusted_valley2).*1/fps);

%cycle extraction

%start from the first peak to last peak

first_peak = indexes_pea(2);

last_peak = indexes_pea(end);

adjusted_peak = indexes_pea(indexes_pea>=first_peak&indexes_pea<=last_peak);

%selecting valleys between peaks

adjusted_valley = indexes_val(indexes_val>first_peak&indexes_val<last_peak);

figure(2)

plot(t(first_peak:last_peak),cont_ana(first_peak:last_peak))

hold on

scatter(t(adjusted_valley),cont_ana(adjusted_valley))

scatter(t(adjusted_peak),cont_ana(adjusted_peak))

hold off

%Timing

A_cycle_time = diff(adjusted_peak).*1/fps;

A_contraction_time =(adjusted_valley-adjusted_peak(1:end-1)).*1/fps;

A_relaxation_time = (adjusted_peak(2:end)-adjusted_valley).*1/fps;

%Contraction Velocity

figure(3)

yyaxis left

plot(t,cont_ana,'LineWidth',1.5)

ylabel('Heart slice movement (a.u)','FontSize',12)

box off

hold on

yyaxis right

plot(t,speed,'LineWidth',1.5)

ylabel('movement speed (a.u)/sec','FontSize',12)

xlabel('Time (s)','FontSize',12)

% axis([0 6 -inf inf])

% axis([1.667 2.5 -inf inf])

hold off

%=========================================================================%

%=========================================================================%

figure(4)

yyaxis left

plot(t,cont_ana,'LineWidth',1.5)

ylabel('Heart slice movement (a.u)','FontSize',12)

set(gca,'ycolor','k') ;

box off

hold on

yyaxis right

plot(t,speed,'--','LineWidth',1.5)

ylabel('movement speed (a.u)/sec','FontSize',12)

xlabel('Time (s)','FontSize',12)

% axis([0 6 -inf inf])

% axis([1.667 2.5 -inf inf])

hold off

set(gca,'ycolor','k') ;

set(gca,'FontSize',10);

legend('Heart slice movement','movement speed')

figure(11)

**References**

[1] N. Nam-Trung, W. Steven, and S. Seyed Ali Mousavi, *Fundamentals and Applications of Microfluidics, Third Edition*. Artech, 2019, p. 1.
