# Supplementary material for: Biomimetic cardiac tissue culture model (CTCM) to emulate cardiac physiology and pathophysiology ex vivo
Source: Commun Biol. 2022 Sep 9;5:934. doi: 10.1038/s42003-022-03919-3 (PMC9463130; doi:10.1038/s42003-022-03919-3)
Supplement: Supplementary file 7 — reporting summary [file 42003_2022_3919_MOESM7_ESM.pdf]

nature research

Corresponding author(s): Tamer M A Mohamed

Last updated by author(s): 08/11/2022

Reporting Summary

Nature Research wishes to improve the reproducibility of the work that we publish. This form provides structure for consistency and transparency in reporting. For further information on Nature Research policies, see our Editorial Policies and the Editorial Policy Checklist.

Statistics

For all statistical analyses, confirm that the following items are present in the figure legend, table legend, main text, or Methods section.

n/a	Confirmed	
☐	☒	The exact sample size (*n*) for each experimental group/condition, given as a discrete number and unit of measurement
☐	☒	A statement on whether measurements were taken from distinct samples or whether the same sample was measured repeatedly
☐	☒	The statistical test(s) used AND whether they are one- or two-sided<br>*Only common tests should be described solely by name; describe more complex techniques in the Methods section.*
☐	☒	A description of all covariates tested
☐	☒	A description of any assumptions or corrections, such as tests of normality and adjustment for multiple comparisons
☐	☒	A full description of the statistical parameters including central tendency (e.g. means) or other basic estimates (e.g. regression coefficient) AND variation (e.g. standard deviation) or associated estimates of uncertainty (e.g. confidence intervals)
☐	☒	For null hypothesis testing, the test statistic (e.g. *F*, *t*, *r*) with confidence intervals, effect sizes, degrees of freedom and *P* value noted<br>*Give P values as exact values whenever suitable.*
☒	☐	For Bayesian analysis, information on the choice of priors and Markov chain Monte Carlo settings
☒	☐	For hierarchical and complex designs, identification of the appropriate level for tests and full reporting of outcomes
☒	☐	Estimates of effect sizes (e.g. Cohen's *d*, Pearson's *r*), indicating how they were calculated

Our web collection on statistics for biologists contains articles on many of the points above.

Software and code

Policy information about availability of computer code

Data collection	We mainly used MatLab software platform for coding. Data collection for imaging on Cytation was collected using Gen5.05 software. All sequencing data were collected using Illumina software for Novaseq.
Data analysis	All custom computer codes described in the manuscript are included in the supplementary file named "supplementary Code" and deposited at Zenodo: https://doi.org/10.5281/zenodo.7023518

For manuscripts utilizing custom algorithms or software that are central to the research but not yet described in published literature, software must be made available to editors and reviewers. We strongly encourage code deposition in a community repository (e.g. GitHub). See the Nature Research guidelines for submitting code & software for further information.

Data

Policy information about availability of data

All manuscripts must include a data availability statement. This statement should provide the following information, where applicable:

- Accession codes, unique identifiers, or web links for publicly available datasets
- A list of figures that have associated raw data
- A description of any restrictions on data availability

The authors declare that all data supporting the findings of this study are available within the paper and its supplementary information files. Please supplementary source data excel file for the RNAseq data and supplementary codes file for the computer codes used in analyses. RNAseq data was deposited at GEO with accession number GSE211110. Software code was deposited at Zenodo: https://doi.org/10.5281/zenodo.7023518

Field-specific reporting

Please select the one below that is the best fit for your research. If you are not sure, read the appropriate sections before making your selection.

☒ Life sciences ☐ Behavioural & social sciences ☐ Ecological, evolutionary & environmental sciences

For a reference copy of the document with all sections, see nature.com/documents/nr-reporting-summary-flat.pdf

Life sciences study design

All studies must disclose on these points even when the disclosure is negative.

Sample size	power analyses were performed to choose the group sizes which will provide >80% power to detect a 10% absolute change in the parameter with a 5% Type I error rate.
Data exclusions	No data excluded from the analysis
Replication	All attempts at replication were successful as indicated by the low standard error between replicates. Biological and technical replicates were used for all experiments to ensure reproducibility
Randomization	Tissue slices were randomized before experiments.
Blinding	All analyses were blinded with regard to conditions, and samples were decoded only after all data were analyzed.

Reporting for specific materials, systems and methods

We require information from authors about some types of materials, experimental systems and methods used in many studies. Here, indicate whether each material, system or method listed is relevant to your study. If you are not sure if a list item applies to your research, read the appropriate section before selecting a response.

Materials & experimental systems

n/a	Involved in the study	
☐	☒	Antibodies
☒	☐	Eukaryotic cell lines
☒	☐	Palaeontology and archaeology
☐	☒	Animals and other organisms
☒	☐	Human research participants
☒	☐	Clinical data
☒	☐	Dual use research of concern

Methods

n/a	Involved in the study	
☒	☐	ChIP-seq
☒	☐	Flow cytometry
☒	☐	MRI-based neuroimaging

Antibodies

Antibodies used	The primary antibodies (1:200 dilution in 1% BSA) (Connexin 43 (Abcam; #AB11370), NFATC4 (Abcam; #AB99431) and Troponin-T (Thermo Scientific; #MA5-12960), were added to the section for 90 minutes, followed by the secondary antibodies (1:200 dilution in 1% BSA) Anti-mouse Alexa Fluor 488 (Thermo Scientific; #A16079), Anti-rabbit Alexa Fluor 594 (Thermo Scientific; #T6391)
Validation	We validated all antibodies in our hands using cell lines overexpressing the protein using viral construct as positive controls and negative control

Animals and other organisms

Policy information about studies involving animals; ARRIVE guidelines recommended for reporting animal research

Laboratory animals	Yorkshire pigs (3 months old) (Female) weighing 25-35 kg
Wild animals	The study did not involve wild animals
Field-collected samples	The study did not involve field-collected samples
Ethics oversight	Animal studies were performed in accordance with the University of Louisville animal use guidelines, and the protocols were approved by the Institutional Animal Care and Use Committee (IACUC) and were accredited by the Association for Assessment and Accreditation of Laboratory Animal Care.

Note that full information on the approval of the study protocol must also be provided in the manuscript.
